# FRIENDSHIP IS IN THE DETAILS: CO-CONSTRUCTION OF RELATIONSHIPS AMONG PERSONS WITH DEMENTIA IN LONG-TERM CARE

**DOI:** 10.1093/geroni/igac059.1223

**Published:** 2022-12-20

**Authors:** Pamela Saunders

**Affiliations:** Georgetown University, Washington, District of Columbia, United States

## Abstract

Friendships have been linked to psychological and emotional wellbeing and better physical functioning in older adults. Conversely, negative consequences (e.g., depression) are associated with losing friendships and shrinking social networks. While cognitive decline might be a limiting factor for persons with dementia (PWD) to establish friendships, this has not been proven in the literature. This paper reports on 20 interactions between PWD collected during the Friendship Study (de Medeiros et al. 2011), an ethnographic study of friendship in long-term care (LTC). Participants are male and female residents in an LTC community. Diagnoses range from mild to severe Alzheimer’s disease. Conversational interactions were transcribed and coded for linguistic and discursive devices signaling friendly interactions. Findings reveal that friendships are co-constructed by PWD using 4 primary linguistic discursive devices, including topic (meals, religion, medication, furniture, directions, baking), co-constructed narrative, repetition, and alignment. Implications for future research on friendship among PWD are discussed.

